# Pitfalls in Coagulation Testing: A Pediatric Case of Lupus Anticoagulant Hypoprothrombinemia Syndrome Following Enterocolitis

**DOI:** 10.7759/cureus.92692

**Published:** 2025-09-19

**Authors:** Taiki Homma, Takuya Kamio, Kimihiko Oishi, Kagehiro Amano, Masaharu Akiyama

**Affiliations:** 1 Department of Pediatrics, The Jikei University School of Medicine, Tokyo, JPN; 2 Department of Laboratory Medicine, Tokyo Medical University, Tokyo, JPN

**Keywords:** adenovirus infection, child, hypoprothrombinemia, interference, lupus anticoagulant

## Abstract

In children, the lupus anticoagulant (LA) is transiently produced after infection and rarely causes bleeding tendency, known as LA-hypoprothrombinemia syndrome (LAHPS). In this case report, we describe a child presenting with difficulty standing and walking after enterocolitis and complicated false coagulation abnormalities. Prolonged prothrombin time and activated partial thromboplastin time (APTT); decreased factors II, VIII, IX, XI, and XII; and inhibitors for factors VIII and IX were detected. On the basis of such findings as inhibitor pattern in the APTT cross-mixing test and positivity for serum LA, LAHPS was diagnosed. Physicians should be aware of the interference by LA with phospholipid-based coagulation tests, leading to the misinterpretation of the unusual coagulation abnormalities in LAHPS.

## Introduction

Lupus anticoagulant (LA), an antiphospholipid antibody, increases the risk of thrombosis, as observed in antiphospholipid syndrome [1]. LA hypoprothrombinemia syndrome (LAHPS) disorder is characterized by coagulation factor II activity being reduced owing to acquired autoantibodies against prothrombin [2]. The first documented case of LAHPS was reported by Rapaport et al. in 1960 in a child with systemic lupus erythematosus (SLE) [3]. With fewer than 100 reported cases [4], LAHPS remains an uncommon condition [5,6]. Infections can transiently induce LA production in children, who are typically asymptomatic. However, in pediatric cases of LAHPS, bleeding of various levels of severity can paradoxically occur rather than thrombosis, which occurs in typical LAHPS [2]. We report on a child who developed coagulopathy with a bleeding tendency after adenovirus gastroenteritis, in whom the APTT cross-mixing test was useful for rapidly diagnosing LAHPS.

## Case presentation

**Figure 1:**
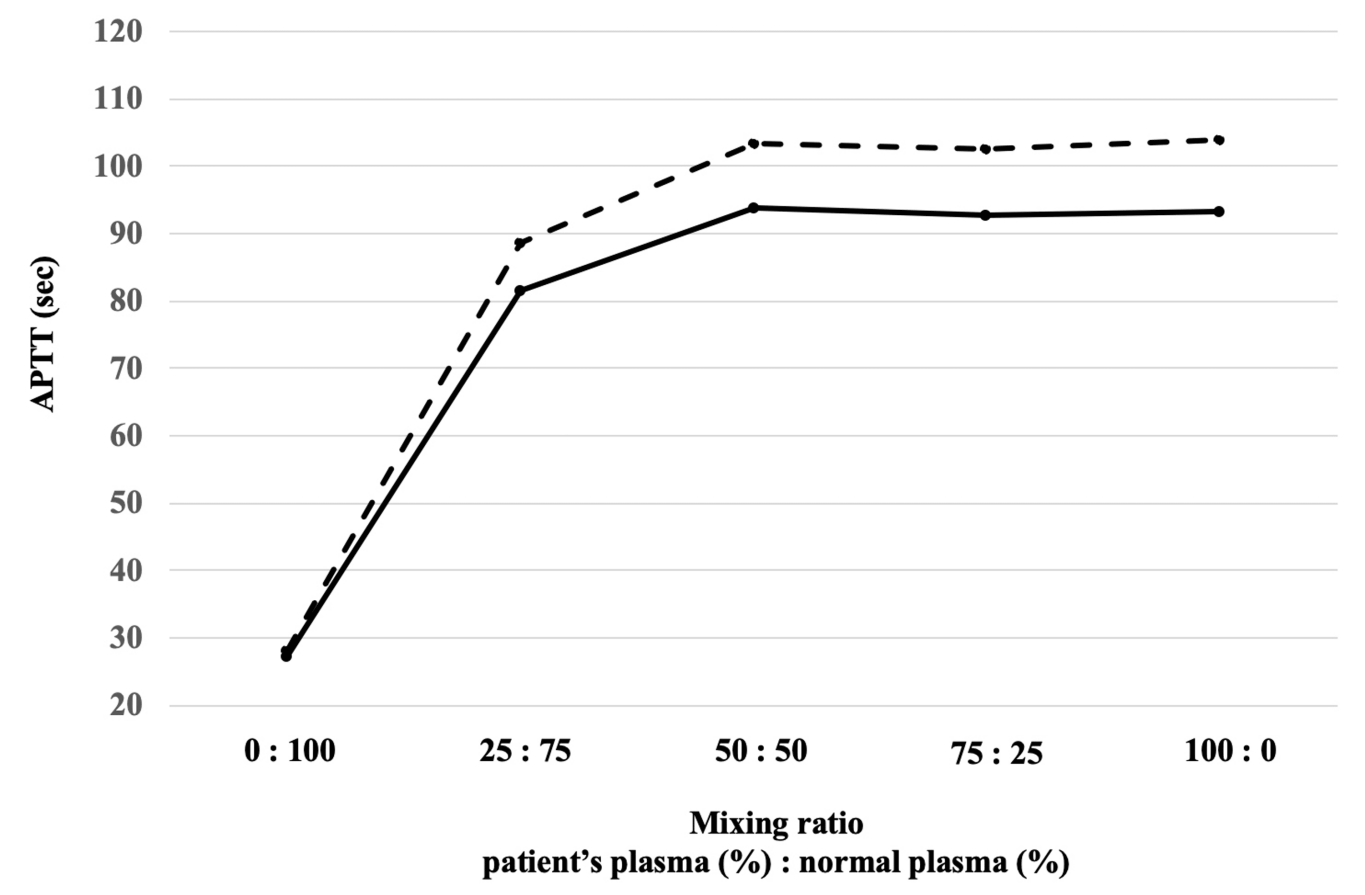
The activated partial thromboplastin time (APTT) cross-mixing test. Five samples involving mixtures of normal plasma and the patient’s plasma at various ratio were used for the APTT cross-mixing test. These samples were measured immediately after mixing (solid line) or after two hours of incubation at 37 °C (dotted line). In the present case, the test showed no dissociation between the immediate and delayed phases, and both the APTT values on the vertical axis and the patient’s plasma ratio on the horizontal axis exhibited a convex pattern, consistent with an inhibitor pattern.

**Figure 2:**
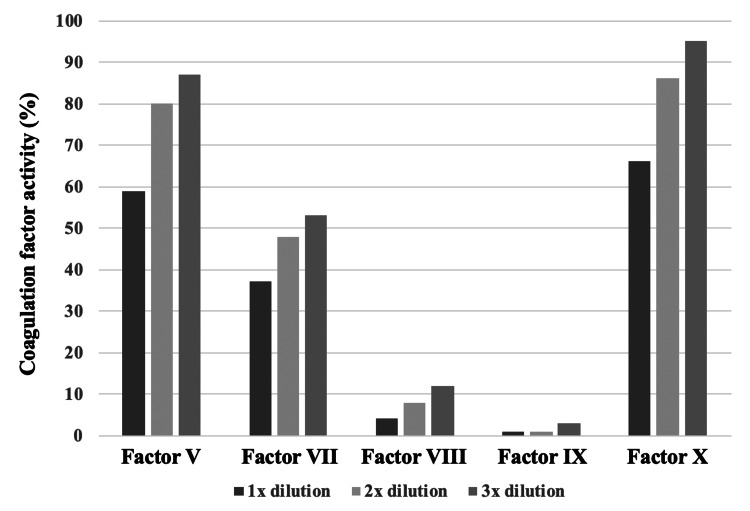
Measurements with dilution of factors V, VII, VIII, IX, and X. Coagulation factors V, VII, VIII, IX, and X were not measurable without dilution. Measurements with 1x, 2x, and 3x dilution showed 59%, 80%, and 87% in factor V; 37%, 48%, and 53% in factor VII; 4%, 8%, and 12% in factor VIII; <1%, <1%, and 2% in factor IX; and 66%, 86%, and 95% in factor X, respectively.

A three-year-old girl who presented with fever, vomiting, and diarrhea was subsequently diagnosed with enterocolitis, which resolved within a few days. However, two weeks later, owing to difficulty in standing and walking due to left knee pain for several days, she was admitted to the Jikei Katsushika Medical Center. Her medical and family histories were unremarkable. Physical examination revealed swelling and warmth in the left knee joint, along with multiple purpuric lesions on the lower extremities. Blood tests showed a normal platelet count: however, coagulation studies showed a markedly prolonged prothrombin time (PT) of 32% (reference range: 70%-130%), a PT international normalized ratio of 2.15 (reference range: 0.9-1.15), and an activated partial thromboplastin time (APTT) of 108.5 seconds (reference range: 24-36 seconds) (Table 1). A short-T1 inversion recovery magnetic resonance image showed a high-signal area behind the left knee, suggesting edematous changes. The APTT mixing test showed an inhibitor pattern (Figure 1). The results of a coagulation factor activity test performed on admission were obtained on the hospital day 6 (Table 1): factor II activity was 8%; factors XI and XII were less than 3%; and factors V, VII, VIII, IX, and X were not measurable without dilution but became measurable with dilution (Figure 2). Inhibitors for factors VIII and IX were detected at 8 and 10 Bethesda units/mL, respectively. The LA/dilute Russell’s viper venom time assay ratio was 1.6 (reference range: 0-1.2).

**Table 1:** Sequential data of coagulation tests. ab2GPI, anti-b2-glycoprotein I; APTT, activated partial thromboplastin time; dRVVT, dilute Russell’s viper venom time; FDP, fibrin degradation products; IgG, immunoglobulin G; INR, international normalized ratio

Parameters	Reference range	On admission	4 weeks later
Prothrombin time (%)	≥ 70	32	100
Prothrombin time INR		2.15	0.94
APTT (sec)	24-36	108.5	46.1
Fibrinogen (mg/dL)	150-400	531	278
Antithrombin (%)	80-115	136	125
Fibrin degradation products (mg/mL)	0-5	2	2
D-dimer (mg/mL)	0-1	0.7	0.6
Factor II (%)	75-135	8	96
Factor V (%)	70-135	59	-
Factor VII (%)	75-140	37	-
Factor VIII (%)	60-150	4	87
Factor IX (%)	70-130	< 1	49
Factor X (%)	75-140	66	-
Factor XI (%)	75-145	< 3	87
Factor XII (%)	50-150	< 3	63
Factor XIII (%)	70-140	78	-
von Willebrand factor antigen (%)	50-155	130	-
von Willebrand factor activity (%)	60-170	122	-
Protein C activity (%)	64-146	297	112
Protein S activity (%)	56-126	243	82
Factor VIII inhibitor (Bethesda units/mL)	negative	8	negative
Factor IX inhibitor (Bethesda units/mL)	negative	10	negative
Lupus anticoagulant/dRVVT	0-1.2	1.6	1.1
anticardiolipin IgG (U/mL)	0-9	7	5.1
ab2GPI IgG (U/mL)	0-3.4	1.2	1.2

Upon admission, serological testing showed an adenovirus antibody titer (complement fixation) of 64 (baseline: 0-3) following the resolution of enterocolitis. However, antibody levels could not be assessed during the acute phase of enterocolitis. Antibodies against phosphatidylserine and prothrombin were not ordered by the physicians.

On the basis of the clinical course and laboratory findings, LAHPS secondary to enterocolitis, likely caused by an adenovirus infection, was diagnosed. Because the arthralgia and purpura had remained unchanged, the patient was treated with oral prednisolone (1.0 mg/kg/day) for seven days. Because PT and APTT were normalized and the symptoms had resolved, the patient was discharged on hospital day 11. Four weeks after discharge, inhibitors of factors VIII and IX were not detected; coagulation factor abnormalities, including factor II activity, had resolved; and LA was no longer detectable (Table 1).

## Discussion

Diagnosing LAHPS on the basis of routine blood tests can be challenging owing to artifactual abnormalities in coagulation parameters. Although Ieko et al. proposed three criteria for diagnosing LAHPS [2], i.e., LA positivity, reduced coagulation factor II activity, and positive antithrombin antibody, no internationally agreed-upon criteria or diagnostic guidelines exist. The index of suspicion for LAHPS should be high in patients presenting with prolonged APTT and a spectrum of bleeding symptoms, ranging from minor to severe hemorrhage, if there has been no medical history or family history of coagulation disorders. From 1996 through 2019, 86 pediatric cases of LAHPS have been reported, including 40 cases from Japan [7]. The clinical characteristics of these children with LAHPS were not sex-specific, but the median age was 6.3 years, with an age range of 0.8 to 15 years. The most common underlying diseases were infection (62.6%) and SLE (24.4%). Viruses reported to cause LAHPS include adenovirus, Epstein-Barr virus, cytomegalovirus, varicella, parvovirus, hepatitis C virus, human immunodeficiency virus, and mycoplasma. Of these viruses, adenovirus was suspected in the present case, and adenovirus infections often precede LAHPS [2]. The most common symptom of LAHPS is minor bleeding (77.4%), but severe bleeding, such as intracranial or gastrointestinal hemorrhage, has been reported in 22.6% of patients. Laboratory findings of these patients included prolonged APTT (100%), decreased coagulation activity of factor II (100%), decreased coagulation activity of other coagulation factors (72.1%), detection of LA (100%), anticardiolipin (aCL) immunoglobulin G (42.6%), and aCL/β2-glycoprotein I (17.0%) [7]. In the present patient, we observed a much longer prolongation of PT and APTT; decreased activity of coagulation factors II, VIII, IX, XI, and XII; positive inhibitors of coagulation factors VIII and IX; and the presence of LA.

No standard management for LAHPS has been established. A review of previously reported cases of LAHPS has found that 53.8% of patients required no treatment, 22.5% required supportive care, and 36.3% required immunosuppressive agents (corticosteroids only, 20.0%; others, 16.3%) [7]. Factors that differed significantly between patients who required or did not require immunosuppressive agents were age, level of coagulation activity of factor II, SLE as an underlying disease, severe bleeding, and positive rates of aCL immunoglobulin G and aCL/β2-glycoprotein I [2]. The present patient was at first maintained under clinical surveillance only, but joint pain and purpura did not improve. The patient received oral prednisolone (1.0 mg/kg/day) for seven days, based on a previous report [4], resulting in normalization of PT and APTT, and the resolution of various coagulation abnormalities.

The patient's phospholipid-based laboratory findings, including inability to measure factors VIII, IX, XI, and XII without dilution, positivity for inhibitors of factor VIII and IX, and high activity of protein S and protein C, are suggestive of interference by LA. When PT or APTT is prolonged, an APTT cross-mixing test is performed to differentiate the coagulation factor inhibitor pattern from the coagulation factor-deficient pattern. This test measures the coagulation time of mixed plasma, in which the patient's plasma and normal plasma obtained from a healthy donor are mixed in various ratios, and the results are graphed for visual evaluation [8]. Measurements are made either immediately after mixing (immediate type) or after 2 hours of incubation at 37°C (delayed type). In the coagulation factor deficiency pattern, both immediate and delayed forms show a downward convex pattern, whereas in the inhibitor pattern, such as LA-positive, both immediate and delayed forms show a similar linear or upward convex pattern [9]. In the present patient, the APTT cross-mixing test showed no dissociation between the immediate and delayed measurement types, and both the APTT on the vertical axis and the patient plasma ratio on the horizontal axis were convex, suggesting an inhibitor pattern and leading to a rapid and correct understanding of the pathogenesis of the multiple abnormal data of coagulation tests.

## Conclusions

A lack of understanding of this phenomenon-phospholipid-based laboratory findings interfered by LA-can lead to the misinterpretation of the unusual coagulation abnormalities in LAHPS, and might result in diagnostic confusion and inappropriate treatment decisions. An accurate diagnosis is crucial for guiding treatment selection. The present case highlights the utility of the APTT cross-mixing test in promptly identifying and overcoming the diagnostic challenges posed by LAHPS.
